# Stabilizing metastable tetragonal HfO_2_ using a non-hydrolytic solution-phase route: ligand exchange as a means of controlling particle size†
†Electronic supplementary information (ESI) available. See DOI: 10.1039/c6sc01601d


**DOI:** 10.1039/c6sc01601d

**Published:** 2016-05-03

**Authors:** Gregory R. Waetzig, Sean W. Depner, Hasti Asayesh-Ardakani, Nicholas D. Cultrara, Reza Shahbazian-Yassar, Sarbajit Banerjee

**Affiliations:** a Department of Chemistry and Department of Materials Science and Engineering , Texas A&M University , College Station , TX 77845-3012 , USA . Email: banerjee@chem.tamu.edu; b Department of Chemistry , University at Buffalo , The State University of New York , Buffalo , New York 14260-3000 , USA; c Department of Mechanical Engineering-Engineering Mechanics , Michigan Technological University , Houghton , Michigan 49933-1295 , USA; d Department of Physics , University of Illinois at Chicago , Chicago , Illinois 60607-7059 , USA; e Department of Mechanical and Industrial Engineering , University of Illinois at Chicago , Chicago , Illinois 60607-7059 , USA

## Abstract

There has been intense interest in stabilizing the tetragonal phase of HfO_2_ since it is predicted to outperform the thermodynamically stable lower-symmetry monoclinic phase for almost every application where HfO_2_ has found use by dint of its higher dielectric constant, bandgap, and hardness. However, the monoclinic phase is much more thermodynamically stable and the tetragonal phase of HfO_2_ is generally accessible only at temperatures above 1720 °C. Classical models comparing the competing influences of bulk free energy and specific surface energy predict that the tetragonal phase of HfO_2_ ought to be stable at ultra-small dimensions below 4 nm; however, these size regimes have been difficult to access in the absence of synthetic methods that yield well-defined and monodisperse nanocrystals with precise control over size. In this work, we have developed a modified non-hydrolytic condensation method to precisely control the size of HfO_2_ nanocrystals with low concentrations of dopants by suppressing the kinetics of particle growth by cross-condensation with less-reactive precursors. This synthetic method enables us to stabilize tetragonal HfO_2_ while evaluating ideas for critical size at which surface energy considerations surpass the bulk free energy stabilization. The phase assignment has been verified by atomic resolution high angle annular dark field images acquired for individual nanocrystals.

## Abstract 2

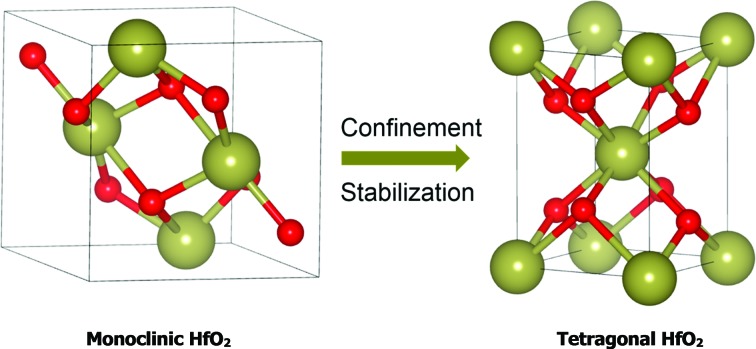
A non-hydrolytic condensation route allows for precise control over the size distribution of HfO_2_ nanocrystals and enables the stabilization of the tetragonal phase of HfO_2_.

## Introduction

A particularly powerful aspect of nanoscience that enables much new functionality derives from the greatly altered phase equilibria obtained upon confining materials to nanoscale dimensions.^1^–^4^ The increased contributions from surface free energy terms can outweigh bulk free energy considerations and enable the stabilization of crystalline phases under ambient conditions that can otherwise only be stabilized at high temperatures and pressures. As a few notable examples of such metastable phases and their interesting properties: a metallic 1T phase is stabilized upon exfoliating the bulk semiconducting 2H-phases of MoS_2_ and WS_2_ to few-layered sheets^5^,^6^ and shows greatly improved activity as a catalyst for hydrogen evolution; a γ-MoC phase is stabilized in preference to β-Mo_2_C for nanostructures of molybdenum carbide and also exhibits promising reactivity for hydrogen evolution;^7^ the tetragonal phase of ZrO_2_ is stabilized in preference to the monoclinic phase for particles below a critical size of *ca.* 30 nm and is used for the transformation toughening of ceramics;^8^,^9^ and a metastable Li_*x*_FePO_4_ phase is stabilized upon electrochemically delithiating LiFePO_4_ nanoparticles at high rates within a Li-ion battery and mitigates the need to go through a much slower nucleation and growth process that would result in phase segregation of LiFePO_4_ and FePO_4_.^10^,^11^ Stabilizing metastable structures requires careful control of particle size and the kinetics of crystallization processes. Low-temperature solution-phase routes for defining structural frameworks are particularly well-suited for preparing kinetically trapped metastable structures since the energetics of these reactions are often too low to allow for coarsening of grain size or overcoming shallow valleys in the potential energy landscape.^12^–^15^ In this work, we demonstrate a cross-coupling ligand exchange method for controlling the particle size in the non-hydrolytic sol–gel condensation growth of HfO_2_ nanocrystals. Precise control of the size of HfO_2_ nanocrystals enables unequivocal determination of the critical size required to stabilize the tetragonal phase and provides access to well-defined crystals of this technologically important metastable structure that is otherwise stable only above *ca.* 1720 °C.^16^

Tetragonal ZrO_2_ has been widely accessible for several decades, even without incorporation of dopant atoms, since the critical size at which the surface free energy terms overcome bulk free energy considerations is relatively large, variously estimated to be *ca.* 15–30 nm.^8^,^9^,^17^–^19^ In contrast, stabilization of the tetragonal phase of HfO_2_ is much more challenging since: (a) the bulk free energy stabilization of the monoclinic phase over the tetragonal phase is almost 40% greater for HfO_2_ as compared to ZrO_2_; and (b) the volume expansion accompanying the tetragonal to monoclinic phase transition is much smaller for HfO_2_ (*ca.* 2.7%) as compared to ZrO_2_ (4.0%).^20^,^21^ Estimates of the critical size required to stabilize the tetragonal phase of HfO_2_ vary widely from about 2 to 10 nm but it is clear that this value is substantially smaller than the critical size for ZrO_2_.^16^,^20^,^22^ Several methods for the preparation of nanometer-sized particles of HfO_2_ report the stabilization of at least some fraction of tetragonal HfO_2_; for instance, signatures of the tetragonal phase have been identified in particles obtained by the thermal decomposition of pure Hf(OH)_4_,^16^ the oxidation of metallic Hf nanocrystals,^23^ and a ligand-mediated reaction at the interface of water and oil phases.^24^ However, the polydispersity and relatively poor crystallinity of the particles obtained by these methods implies that the obtained samples almost always contain only minor proportions of tetragonal phases and a clear delineation of a size-dependent phase diagram has thus far not been possible. Several instances of stabilizing tetragonal domains have also been reported for thin films of HfO_2_ prepared by methods such as atomic layer deposition in a low-oxygen environment,^25^ ultra-thin films deposited onto clean Si (100) surfaces by atomic layer deposition,^26^ and ion-beam-assisted deposition in an oxygen deficient ambient.^27^ Again, the films usually contain mixtures of multiple crystalline and amorphous phases and exhibit considerable heterogeneity in terms of the dimensions of individual domains.

The stabilization of tetragonal HfO_2_ is not just an academic curiosity. Indeed, the tetragonal (space group *P*4_2_/*nmc*) phase of HfO_2_ is anticipated to outperform the thermodynamically stable lower-symmetry monoclinic phase (space group *P*2_1_/*c*) for almost every application where HfO_2_ has found use. Perhaps the most important application of HfO_2_ is in gate dielectric stacks as a high-*κ* dielectric because of its resistance to silicidation and silicate formation and its much greater dielectric constant as compared to SiO_2_.^20^,^28^–^30^ First-principles calculations predict a dielectric constant of 18 for monoclinic HfO_2_, which is far surpassed by the value of almost 70 predicted for tetragonal HfO_2_.^31^ The bandgap of the tetragonal phase is also predicted to be larger (*ca.* 6.11 eV) as compared to the monoclinic phase (5.78 eV).^32^ Finally, the tetragonal phase is denser and has been experimentally found to have a higher Knoop hardness.^27^

In comparison to aqueous methods, non-hydrolytic methods, often based on formation of oxo-bridges by elimination of small molecules, provide considerable control of the kinetics of condensation.^14^,^15^,^33^ Such control is imperative to precisely control particle size and morphology when growing nanoparticles. In an early work, the condensation of hafnium alkoxides in benzyl alcohol with the elimination of alkyl ethers yielded monoclinic, but not tetragonal, HfO_2_ nanocrystals.^34^ Solvothermal processing of various hafnium alkoxides yielded varying morphologies of HfO_2_ nanocrystals, again crystallized in the monoclinic phase.^35^ Hyeon and co-workers adapted the alkyl halide elimination route first proposed by Vioux for the growth of nanocrystals and were able to achieve the multigram synthesis of ZrO_2_ nanocrystals as per:^17^,^36^

1 ≡M–X + ≡M–O–R → ≡M–O–M≡ + R–X

**Scheme 1:**
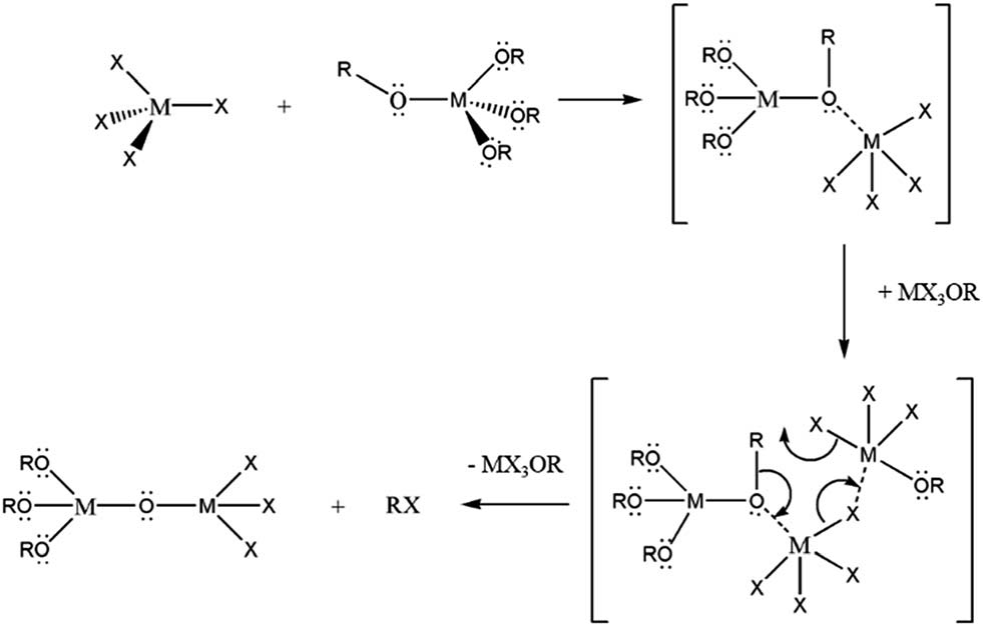
Non-hydrolytic condensation of metal halide and metal alkoxide to form an alkoxo-bridge intermediate. A “catalyst”, MX_3_OR, initiates conversion of the alkoxo-bridge to an oxo-bridge then subsequently eliminates an alkyl halide to form the desired metal oxide-product.

Brus and co-workers extended this method to prepare HfO_2_ and solid-solution Hf_*x*_Zr_1–*x*_O_2_ nanocrystals and established considerable control over the relative Hf : Zr concentrations.^19^ In previous work, we have demonstrated that the R group of the alkoxide ligand allows for substantial tunability of the size of HfO_2_ and ZrO_2_ nanocrystals as well as the relative Hf : Zr ratios of Hf_*x*_Zr_1–*x*_O_2_ nanocrystals.^37^,^38^ These studies as well as past work by Vioux have established that upon reacting M(OR)_4_ and MX_4_ species, substantial ligand exchange takes place to stabilize haloalkoxides such as M(OR)_3_Cl, M(OR)_2_Cl_2_, and M(OR)Cl_3_.^14^,^36^ The first of these three species has been proposed as a catalyst that brings about transformation of an alkoxo-bridge to an oxo-bridge as per the reaction depicted in Scheme 1.

When two different metal precursors are reacted, the composition of the product depends on the relative condensation rates and reactivities. For metals exhibiting comparable reactivity, such as Hf and Zr, the heterocondensation reaction yields solid-solution nanocrystals and can be written as follows:^18^,^19^

2 *m*MX_*n*_ + *n*M′(OR)_*m*_ → M_*m*_M′_*n*_O_*nm*_ + *nm*RX

However, if the rates of homo- and hetero-condensation are vastly different, the products of the faster reaction are obtained preferentially. In this work, we find that adding a much less reactive precursor, either Ce(O^*t*^Bu)_4_ or La(O^i^Pr)_3_, greatly modifies the concentration of the active monomer depicted in Scheme 1 and thereby retards the kinetics of the homocondensation reaction, yielding considerable control over the size of HfO_2_ nanocrystals with only minimal incorporation of La and Ce. This unprecedented control of nanocrystal size allows us to explore ultra-small dimensions and determine the critical size for stabilizing the tetragonal phase of HfO_2_.

## Experimental

### Synthesis

Hafnium(iv) chloride, cerium(iii) chloride, lanthanum(iii) chloride, lanthanum(iii) iso-propoxide, and tri-*n*-octylphosphine oxide (TOPO) were purchased from Strem and used without any further purification. Hafnium(iv) *tert*-butoxide was purchased from Alfa Aesar and used as received. Cerium(iv) *tert*-butoxide was purchased from Gelest and used without further purification. To synthesize the metal oxide nanocrystals, a metal halide and metal alkoxide are mixed in equimolar amounts with *ca.* 10 g of TOPO in a three-neck round bottom flask under an Ar ambient within a glovebox. The total concentration of metal precursors is maintained at 4 mmol in all of the reactions. In all of the reactions, 2 mmol of HfCl_4_ is used as the chloride precursor, whereas the alkoxide precursor is *x* mmol of La(O^i^Pr)_3_ or Ce(O^*t*^Bu)_4_ and 4 – *x* mmol of Hf(O^*t*^Bu)_4_. CeO_2_ nanocrystals were obtained by the reaction of equimolar amounts of CeCl_3_ and Ce(O^*t*^Bu)_4_ as reported in our previous work.^39^ Reducing the amount of the chloride precursor below equimolar amounts solely yields amorphous products as discussed in further detail below.

Briefly, the reaction mixture is heated under an argon ambient on a Schlenk line to *ca.* 60 °C, until the TOPO is melted at which point stirring is initiated. Subsequently, the reaction mixture is heated to 340 °C and kept at this temperature for 2 h. Next, the reaction mixture is cooled to *ca.* 60 °C and solvent/non-solvent washing is performed alternating acetone and hexanes to remove excess TOPO.

### Characterization

A Rigaku Ultima IV diffractometer with a graphite monochromator and a Bruker-AXS D8 Advanced Bragg–Brentano X-ray powder diffractometer, both using Cu Kα radiation (*λ* = 1.5418 Å), were used for characterization of the samples by powder X-ray diffraction. The crystallinity and size distribution of the nanocrystals was evaluated by high-resolution transmission electron microscopy (HRTEM) using JEOL-2010 and FEI Tecnai G2 F20 ST electron microscopes at an operating voltage of 200 kV. Samples for HRTEM analysis were dispersed in hexanes, drop-cast onto 400-mesh carbon-coated copper grids, and allowed to dry in air. The stoichiometry of the nanocrystals was determined by X-ray fluorescence (XRF) measurements, conducted by Oneida Research Services, Inc. in Whitesboro, NY, by inductively coupled plasma-mass spectrometry (ICP-MS) using a Perkin Elmer DRCII instrument, and by energy dispersive X-ray spectroscopy (EDX) on a JEOL JSM-7500F field emission scanning electron microscope (FE-SEM) operated at an accelerating voltage of 20 kV. Samples for ICP-MS analysis were prepared by acid digestion in an aqueous solution of 67% metals grade HNO_3_. Further characterization was performed using atomic resolution high-angle annular dark-field (HAADF) imaging. A probe-corrected JEOL JEM-ARM200CF instrument equipped with a cold field-emission gun operated at 200 kV was used with a convergence angle of 22 mrad. The HAADF inner detector angle was 90 mrad.

## Results

**Fig. 1:**
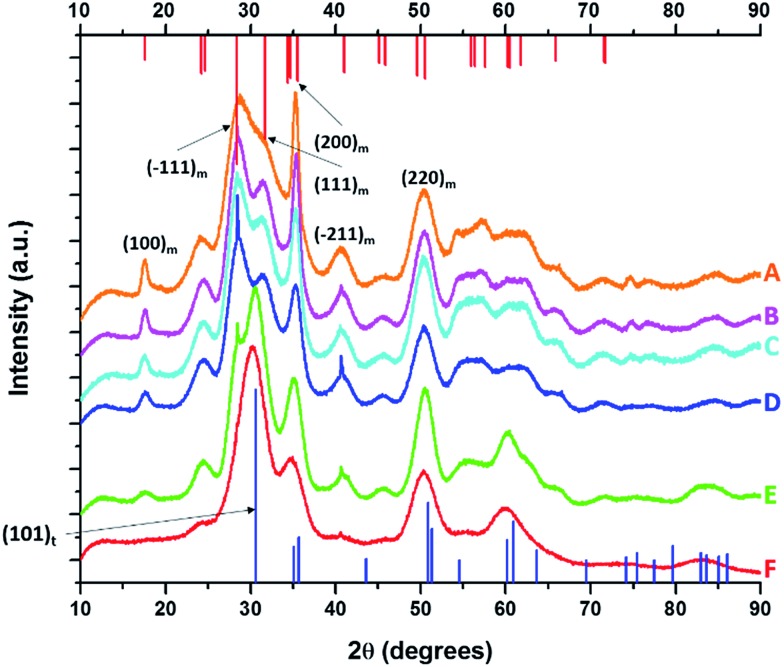
X-ray diffraction patterns of end-member pure HfO_2_ (A) compared to Hf_1–*x*_Ce_*x*_O_2_ nanocrystals prepared by the reaction of HfCl_4_ with varying proportions of Hf(O^*t*^Bu)_4_ and Ce(O^*t*^Bu)_4_. The patterns correspond to detected (and precursor) relative Hf concentrations. The precursors are listed in Table 1. (A) 100% (100%), (B) 99.87% (90%), (C) 99.87% (80%), (D) 99.68% (70%), (E) 99.36% (60%) and (F) 97.64% (50%). Vertical bars indicate positions and relative intensities of reflections expected from JCPDS patterns. Reflections of monoclinic HfO_2_ are indicated in red (JCPDS # 78-0050) and tetragonal HfO_2_ in blue (XRD pattern simulated as described in the text).

Fig. 1 displays XRD patterns for nanocrystals obtained by the reaction of HfCl_4_ with varying proportions of Hf(O^*t*^Bu)_4_ and Ce(O^*t*^Bu)_4_. Table 1 lists the actual Hf and Ce atomic concentrations obtained for the different precursor ratios as well as the primary phase identified from analysis of powder XRD patterns. Energy dispersive X-ray spectroscopy (EDX) was also performed on the Hf_1–*x*_Ce_*x*_O_2_ nanocrystals and provides good agreement with the hafnium and cerium concentrations derived by elemental analysis (Fig. S1 and Table S1, ESI†). Decreasing the concentration of HfCl_4_ below 2 mmol results in the formation of entirely amorphous aggregates and thus Hf : Ce concentrations with excess cerium in the reaction mixture are not further discussed. Lower alkoxide concentrations further yield amorphous products. HfO_2_ nanocrystals prepared by the reaction of Hf(O^*t*^Bu)_4_ and HfCl_4_ are clearly monoclinic (*P*2_1_/*c*) as delineated by the appearance of sharp (200) and (220) reflections (Joint Committee on Powder Diffraction Standards (JCPDS) 78-0050) indexed in Fig. 1A.^37^,^38^ In contrast, CeO_2_ nanocrystals crystallize in a cubic fluorite structure (*Fm*3[combining macron]*m*) with reflections that can be indexed to JCPDS# 34-0394 (Fig. S2, ESI†). As the concentration of Hf is decreased, or in other words, the relative ratio of Ce(O^*t*^Bu)_4_ to Hf(O^*t*^Bu)_4_ is increased, the reflections are broadened indicating a pronounced diminution in size even though elemental analysis data presented in Table 1 suggests very little Ce incorporation. In contrast to the rest of the series, reaction between 2 mmol of HfCl_4_ and 2 mmol of Ce(O^*t*^Bu)_4_ preponderantly yields the tetragonal phase of HfO_2_ characterized by a pronounced (101) reflection centered at 2*θ* = 30.3° (Fig. 1F). The simulated pattern of tetragonal HfO_2_ has been generated using coordinates for a relaxed structure of this phase calculated by Perevalov and co-workers and is depicted in blue in Fig. 1 (a complete structure solution is thus far absent for this phase).^40^ Table S2 in the ESI† lists the atomic coordinates, lattice constants, and angles of the monoclinic and tetragonal phases of HfO_2_ with the latter set of data being derived from the calculation noted above. Careful examination of Fig. 1E, the XRD pattern for the sample prepared with 1.6 mmol of Ce(O^*t*^Bu)_4_, indicates the appearance of a reflection at 2*θ* = 31° that can be attributed to the (101) reflection of the tetragonal phase and suggests that this set of precursors yields a mixture of tetragonal and monoclinic phases. A reflection at 2*θ* = *ca.* 41° in Fig. 1E suggests trace amounts of the monoclinic phase although the tetragonal phase is clearly vastly preponderant.

**Table 1:** Relative amounts of precursors used in the synthesis of HfO_2_ nanocrystals, the atomic percentage of Hf as a function of the total metal content, the detected relative Hf and Ce concentrations, the length of the nanorods determined from statistical analysis of TEM data (the width is invariant at *ca.* 2.5 nm), and the predominant phase determined by powder X-ray diffraction

HfCl_4_ [mmols]	Hf(O-^*t*^But)_4_ [mmols]	Ce(O-^*t*^But)_4_ [mmols]	Precursor Hf concentration [relative at%]	Detected Hf concentration [relative at%]	Detected Ce concentration [relative at%]	Length of nanocrystals [nm]	Phase of nanocrystals
2.0	2.0	0.0	100	100.0	0	12.9 ± 1.8	Monoclinic
2.0	1.6	0.4	90	99.87	0.13	9.5 ± 2.2	Monoclinic
2.0	1.2	0.8	80	99.87	0.13	7.8 ± 1.9	Monoclinic
2.0	0.8	1.2	70	99.68	0.32	5.9 ± 1.0	Monoclinic
2.0	0.4	1.6	60	99.36	0.64	4.4 ± 0.7	Monoclinic/tetragonal
2.0	0	2	50	97.64	2.36	3.1 ± 0.4	Tetragonal
0.0	0.0	4.0	0	0	100	1.5 ± 0.5	Cubic

**Fig. 2:**
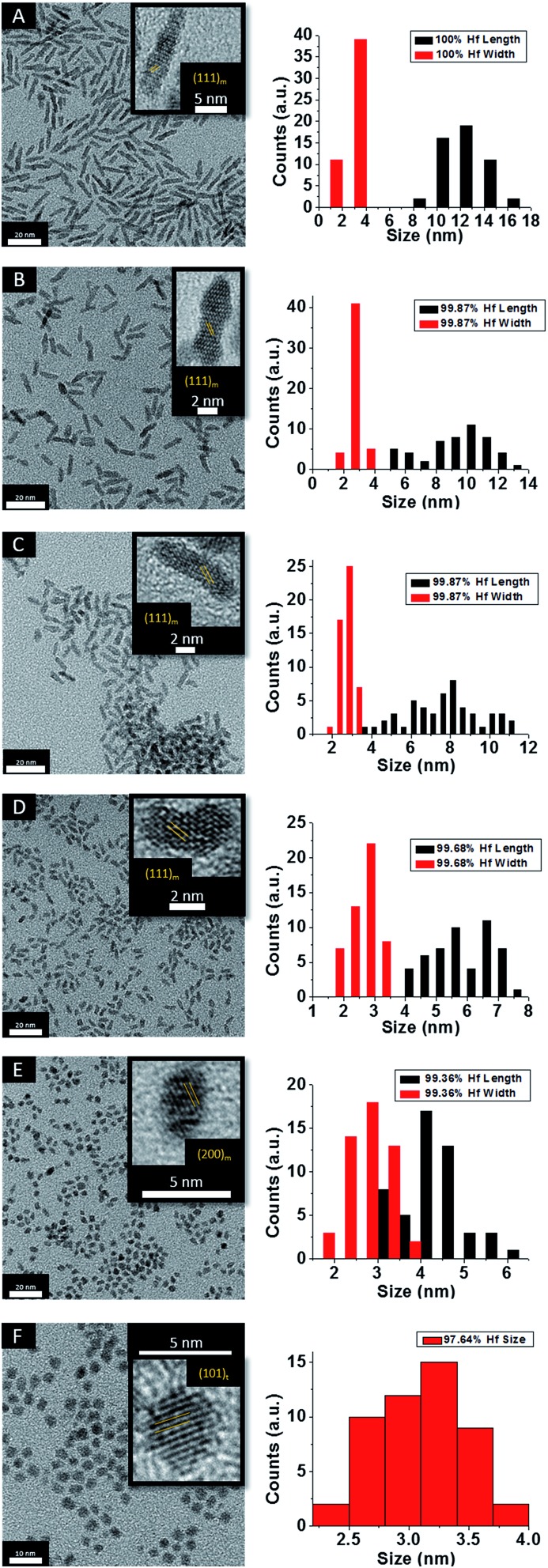
Low-magnification transmission electron microscopy images of HfO_2_ (A) nanocrystals compared to nanocrystals prepared by the reaction of HfCl_4_ with varying proportions of Hf(O^*t*^Bu)_4_ and Ce(O^*t*^Bu)_4_. The precursors and calculated dimensions are listed in Table 1. The detected (and precursor) relative Hf concentrations of (A) 100% (100%), (B) 99.87% (90%), (C) 99.87% (80%), (D) 99.68% (70%), (E) 99.36% (60%), and (F) 97.64% (50%). Insets show HRTEM images. The lattice-resolved images indicate separations between the (111) and (200) lattice planes of the monoclinic phase in the insets to (A) to (E). The separation between the (101) lattice planes of the tetragonal phase is indicated in the inset to (F). The accompanying size distribution histograms are shown to the right of each sample illustrating that the length of the nanorods decreases with increasing concentration of Ce(O^*t*^Bu)_4_ in the synthesis, whereas the width remains relatively constant.

Fig. 2 indicates HRTEM images and size distribution histograms for these nanocrystals. Table 1 lists the dimensions determined by statistical analysis of at least 50 nanocrystals. With increasing concentration of Ce(O^*t*^Bu)_4_ in the reaction mixture, the morphology of the obtained nanostructures evolves from elongated nanorods to quasi-spherical particles. The nanorods preferentially grow along the [100] direction of monoclinic HfO_2_.^38^ As also suggested by the powder XRD data of Fig. 1, the lengths of the Hf_1–*x*_Ce_*x*_O_2_ nanorods are monotonically diminished with reduced concentration of Hf(O^*t*^Bu)_4_ with respect to Ce(O^*t*^Bu)_4_. The average length is decreased from 12.9 ± 1.8 nm for monoclinic nanocrystals grown using only Hf(O^*t*^Bu)_4_ to 3.1 ± 0.4 nm for tetragonal nanocrystals grown using Ce(O^*t*^Bu)_4_ as the only alkoxide precursor. The widths of the Hf_1–*x*_Ce_*x*_O_2_ nanocrystals are not substantially altered and remain around 2.5 ± 0.4 nm. The lattice-resolved HRTEM image in Fig. 2F clearly indicates a lattice separation of 0.292 nm corresponding to the predicted separation between the (101) planes of tetragonal HfO_2_ (Table S2†),^40^ corroborating the stabilization of this metastable phase under these conditions.

**Fig. 3:**
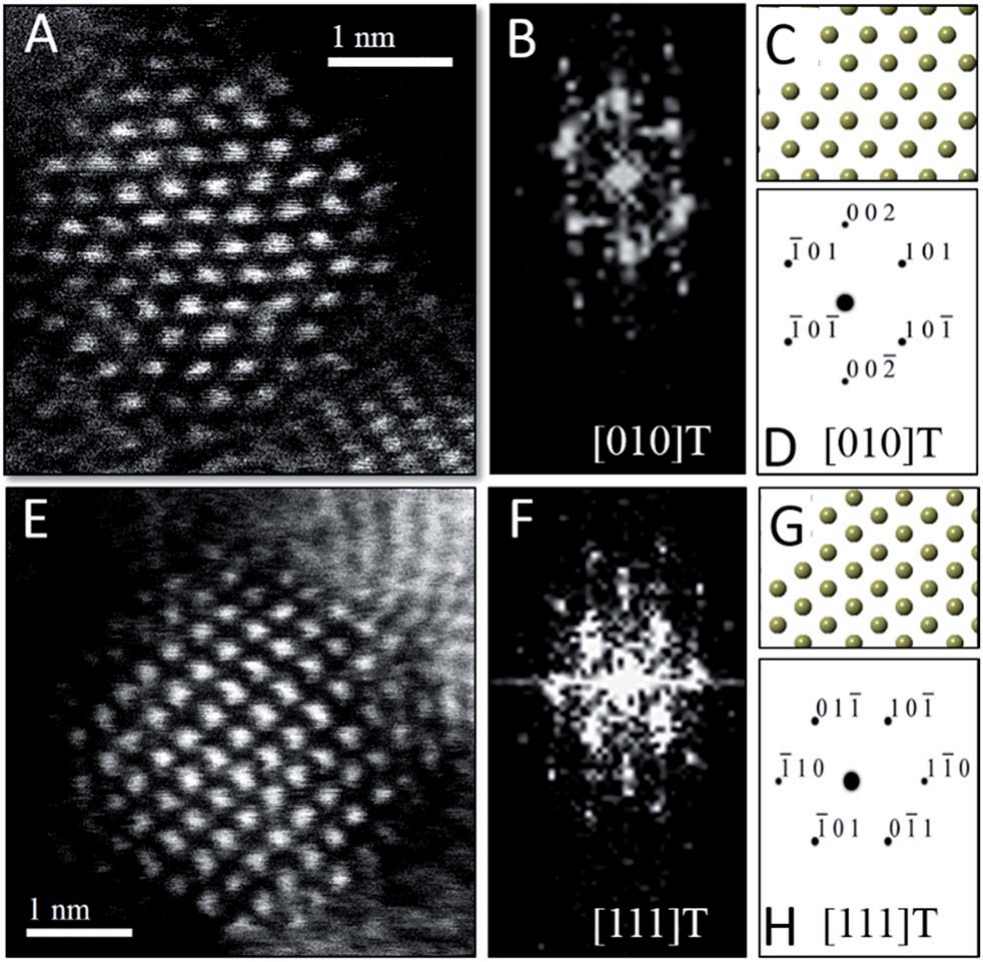
Scanning transmission electron microscopy analysis of Hf_1–*x*_Ce_*x*_O_2_ tetragonal nanocrystals. (A) Atomic-resolution HAADF image and (B) FFT of (A) acquired along the [010] zone axis of the tetragonal structure. (C) Solid sphere model of the tetragonal structure corresponding to the [010] zone axis and (D) simulated diffraction pattern based on (C), both confirming the tetragonal crystal structure of (A). (E) Atomic-resolution HAADF image and (F) FFT of (E) acquired along the [111] zone axis of the tetragonal structure of another nanocrystal. (G) Solid sphere model of the tetragonal structure corresponding to the [111] zone axis and (H) simulated diffraction pattern based on (G).

To verify the phase assignment of Hf_1–*x*_Ce_*x*_O_2_ as being tetragonal, atomic-resolution HAADF scanning transmission electron microscopy (STEM) imaging has been performed using an aberration corrected microscope. Fig. 3 indicates HAADF STEM images and fast Fourier transforms (FFT) acquired for two different nanocrystals along the [010] and [111] zone axes. The simulated diffraction patterns for these zone axis assignments using coordinates of the tetragonal structure are an excellent match to the experimental data (as indicated by the reconstructed solid spheres depiction), providing unequivocal corroboration of the tetragonal crystal structure. Fig. S3A and B† indicate the planes of atoms in the tetragonal unit cell that are indexed in the simulated diffraction patterns (Fig. 3D and H) for each image acquired along a specific zone axis.

**Fig. 4:**
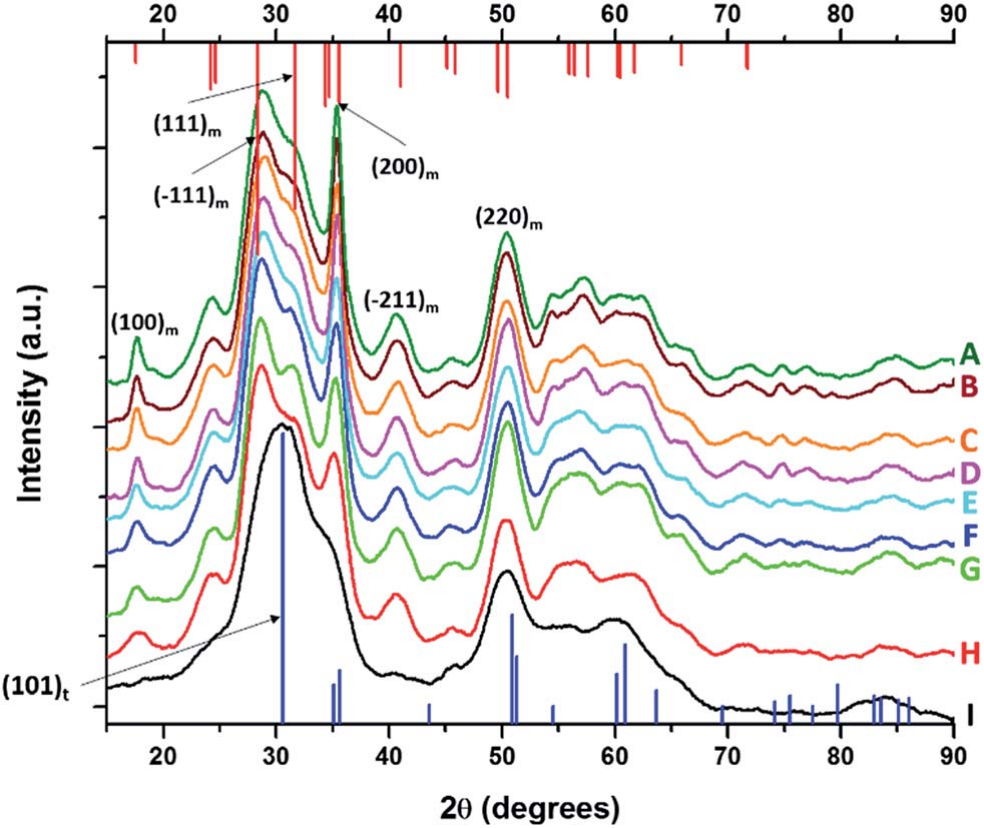
X-ray diffraction patterns of pure HfO_2_ (A) compared to Hf_1–*x*_La_*x*_O_2_ nanocrystals prepared by the reaction of HfCl_4_ with varying proportions of Hf(O^*t*^Bu)_4_ and La(O^i^Pr)_3_. The patterns correspond to detected (and precursor) relative Hf concentrations. The precursors are listed in Table 2. (A) 100% (100%), (B) 99.98% (97.5%), (C) 99.78% (93.75%), (D) 99.56% (87.5%), (E) 98.87% (75%), (F) 98.68% (62.5%), (G) 98.07% (56.25%), (H) 96.46% (52.5%), and (I) 95.27% (50%). Vertical bars indicate positions and relative intensities of reflections expected from JCPDS patterns. Reflections of monoclinic HfO_2_ are indicated in red (JCPDS # 78-0050) and tetragonal HfO_2_ in blue (XRD pattern simulated as described in the text).

Fig. 4 displays powder XRD patterns acquired for nanocrystals obtained by the reaction of HfCl_4_ with varying proportions of Hf(O^*t*^Bu)_4_ and La(O^i^Pr)_3_. Table 2 lists the actual Hf and La atomic concentrations obtained for the different precursor ratios as well as the primary phase identified from analysis of powder XRD patterns. EDX was also performed on Hf_1–*x*_La_*x*_O_2_ nanocrystals and the determined hafnium and lanthanum concentrations are in good agreement with the elemental analysis results (Fig. S4 and Table S3†). Again, upon decreasing the Hf : La ratios such that there is a lower concentration of hafnium precursors as compared to lanthanum precursors, only amorphous aggregates are obtained, and thus we focus our discussion on reaction mixtures with lower concentrations of La(O^i^Pr)_3_ as listed in Table 2. Lower alkoxide concentrations also yield amorphous aggregates. Notably, the homocondensation of LaCl_3_ and La(O^i^Pr)_3_ yields LaOCl nanocrystals crystallized in the matlockite PbFCl-type phase.^41^–^43^

**Table 2:** Relative amounts of precursors used in the synthesis of HfO_2_ nanocrystals, the atomic percentage of Hf as a function of the total metal content, the detected relative Hf and La concentrations, the length of the nanorods determined from statistical analysis of TEM data (the width is invariant at *ca.* 2.5 nm), and the predominant phase determined by powder X-ray diffraction

HfCl_4_ [mmols]	Hf(O-^*t*^But)_4_ [mmols]	La(O-^i^Pro)_3_ [mmols]	Precursor Hf concentration [relative at%]	Detected concentration [relative at%]	Detected Ce concentration [relative at%]	Length of nanocrystals [nm]	Phase of nanocrystals
2.0	2.00	0.00	100	100.0	0.00	12.9 ± 1.8	Monoclinic
2.0	1.90	0.10	97.50	99.98	0.01	11.5 ± 1.26	Monoclinic
2.0	1.75	0.25	93.75	99.78	0.22	10.1 ± 0.89	Monoclinic
2.0	1.50	0.50	87.50	99.56	0.44	9.3 ± 1.46	Monoclinic
2.0	1.00	1.00	75.0	98.87	1.13	8.1 ± 0.98	Monoclinic
2.0	0.50	1.50	62.50	98.68	1.32	7.0 ± 1.02	Monoclinic
2.0	0.25	1.75	56.25	98.07	1.93	5.9 ± 0.92	Monoclinic
2.0	0.10	1.90	52.50	96.46	3.54	4.7 ± 0.79	Monoclinic
2.0	0.00	2.00	50.00	95.27	4.73	3.3 ± 0.42	Tetragonal

Analogous to the above discussion for Ce(O^*t*^Bu)_4_, with increasing concentration of La(O^i^Pr)_3_ in the reaction mixture, the (100) and (200) reflections indexed to the monoclinic phase of pure HfO_2_ are broadened and diminished in intensity suggesting a pronounced diminution in size. Again, although the powder XRD patterns are substantially altered, the elemental analysis results listed in Table 2 indicate very little incorporation of La in HfO_2_. For the sample prepared by the reaction of HfCl_4_ and La(O^i^Pr)_3_, the (100) monoclinic reflection is no longer observed and the (101) tetragonal reflection becomes the most prominent feature in the powder XRD pattern suggesting stabilization of the tetragonal phase of HfO_2_ with modest La doping.

**Fig. 5:**
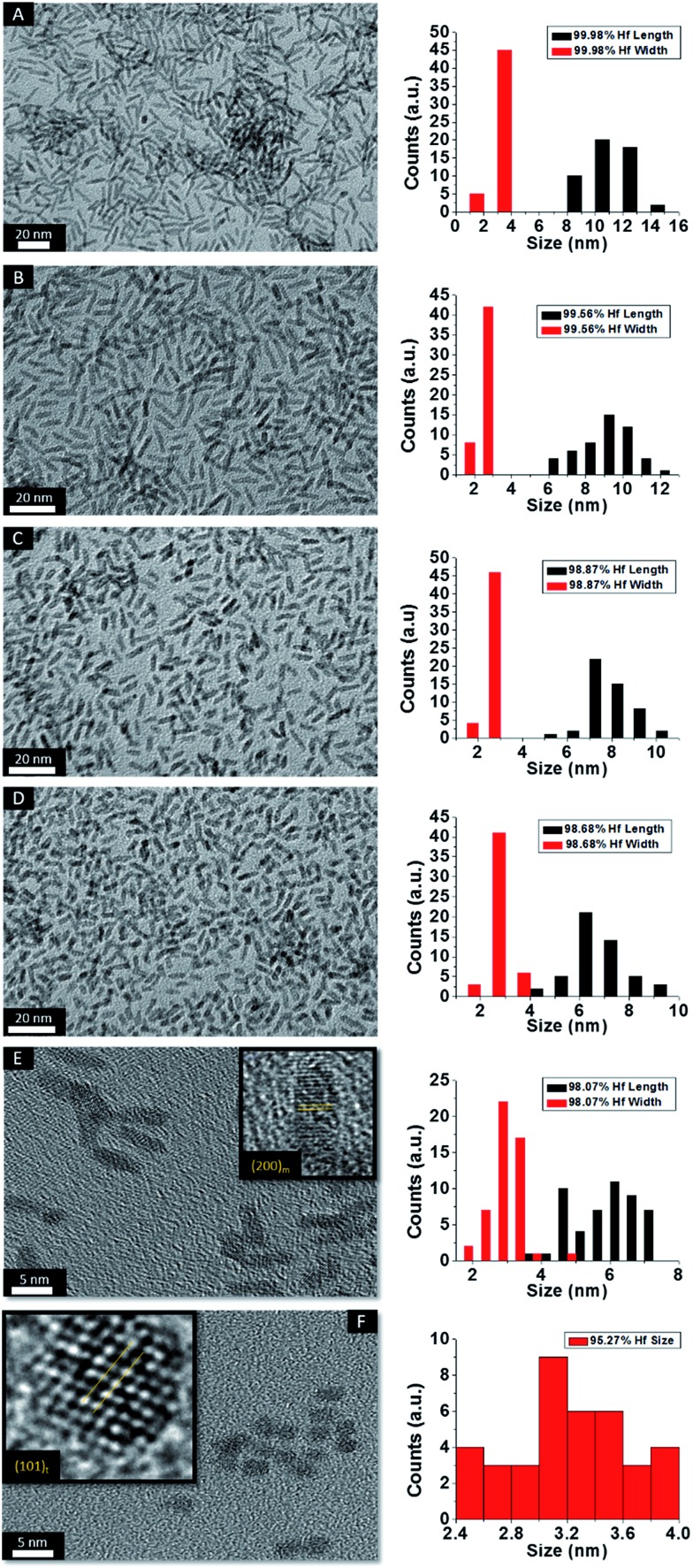
Low-magnification transmission electron microscopy images of nanocrystals prepared by the reaction of HfCl_4_ with varying proportions of Hf(O^*t*^Bu)_4_ and La(O^i^Pr)_3_. The precursors and calculated dimensions are listed in Table 2. The detected (and precursor) relative Hf concentrations of (A) 99.98% (97.5%), (B) 99.56% (87.5%), (C) 98.87% (75%), (D) 98.68% (62.5%), (E) 98.07% (56.25%), and (F) 95.27% (50%). Insets show HRTEM images. The lattice-resolved images indicate separations between the (200) lattice planes of the monoclinic phase in the inset of (E) and the (101) lattice planes of the tetragonal phase in the inset of (F). The accompanying size distribution histograms are shown to the right of each sample illustrating that the length of the nanorods decreases with increasing concentration of La(O^i^Pr)_3_ in the synthesis, whereas the width remains relatively constant.

HRTEM images and size distribution histograms of this set of nanocrystals are shown in Fig. 5 and the relevant dimensions and standard deviations deduced from statistical analysis are listed in Table 2. With increasing concentration of La(O^i^Pr)_3_, the Hf_1–*x*_La_*x*_O_2_ nanocrystals again evolve from elongated nanorods to quasi-spherical nanocrystals. The width of the Hf_1–*x*_La_*x*_O_2_ nanocrystals is relatively unchanged at *ca.* 2.5 ± 0.4 nm for the entire set of samples. However, the length of the nanorods is diminished from 12.9 ± 1.8 nm for monoclinic nanocrystals grown using only Hf(O^*t*^Bu)_4_ to 3.3 ± 0.4 nm for Hf_1–*x*_La_*x*_O_2_ tetragonal nanocrystals grown using La(O^i^Pr)_3_ as the only alkoxide precursor.

**Fig. 6:**
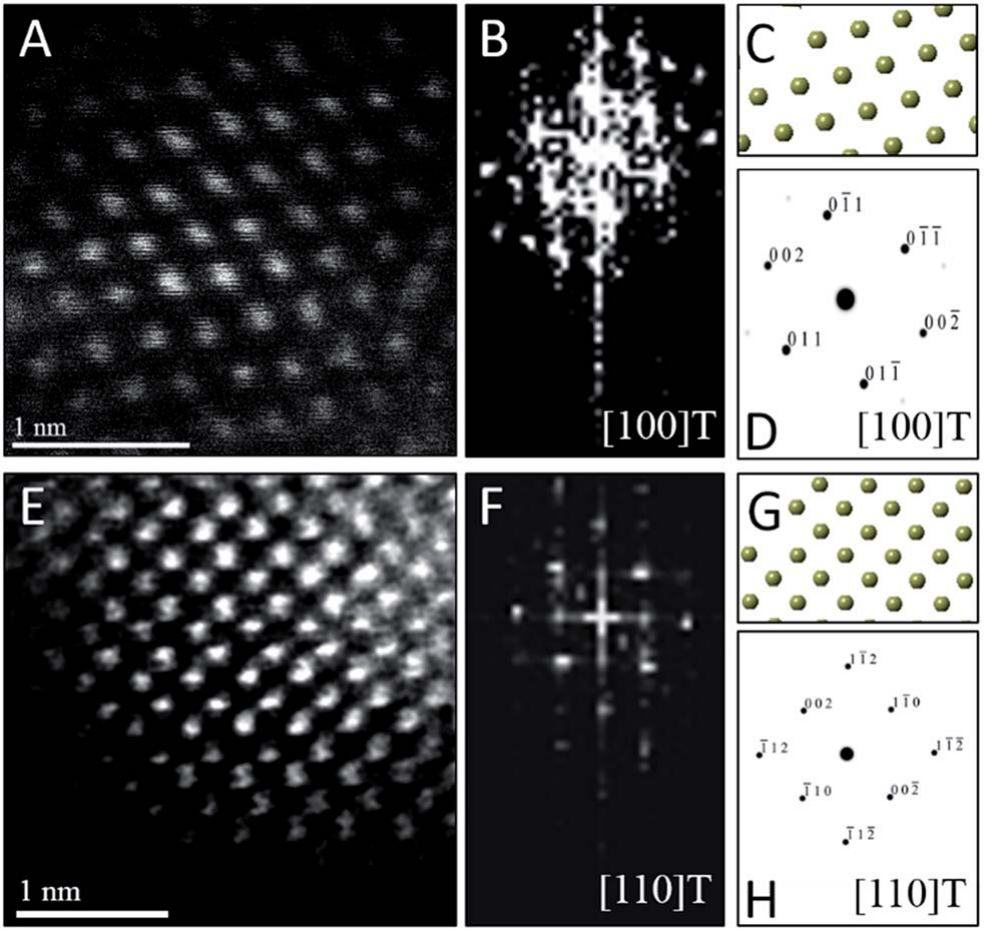
Scanning transmission electron microscopy analysis of individual Hf_1–*x*_La_*x*_O_2_ tetragonal nanocrystals. (A) Atomic-resolution HAADF image and (B) FFT of (A) acquired along the [100] zone axis of the tetragonal structure. (C) Solid sphere model of the tetragonal structure corresponding to the [100] zone axis and (D) simulated diffraction pattern based on (C), both confirming the crystal structure orientation of (A). (E) Atomic-resolution HAADF image and (F) FFT of (E) acquired along the [110] zone axis of the tetragonal structure of another nanocrystal. (G) Solid sphere model of the tetragonal structure corresponding to the [110] zone axis and (H) simulated diffraction pattern based on (G).

To confirm the phase assignment of Hf_1–*x*_La_*x*_O_2_ nanocrystals as being tetragonal, atomic-resolution HAADF STEM images and fast Fourier transforms (FFT) of the HAADF images were acquired and are depicted in Fig. 6. Images were acquired for different nanocrystals along [100] and [110] zone axes. The simulated diffraction patterns are an excellent match to the Fourier transforms in each case and the reconstructed tetragonal lattice (depicted as solid spheres) is entirely superimposable on the STEM image. Fig. S5A and B† indicate the planes of atoms in the tetragonal unit cell that are indexed in the simulated diffraction patterns (Fig. 6D and H). The STEM data further provides unambiguous confirmation of the tetragonal crystal structure.

The preceding discussion illustrates that the addition of Ce(O^*t*^Bu)_4_ and La(O^i^Pr)_3_ leads to a substantial and monotonic diminution of nanocrystal size even though elemental analysis results indicate that relatively low concentrations of Ce and La are actually incorporated within the lattice. At the smallest dimensions, the tetragonal phase of HfO_2_ is stabilized over the monoclinic phase. These results lead to two fundamental questions regarding the mechanistic basis for the size control achieved by addition of La and Ce precursors and the origin of the altered phase stability.

## Discussion

To understand the origin of the size control achieved by the addition of Ce(O^*t*^Bu)_4_ and La(O^i^Pr)_3_, it is important to consider the overall equation depicted in eqn (1), which results in formation of an oxo-bridge with the elimination of an alkyl halide, as well as the catalytic scheme proposed by Vioux shown in Scheme 1.^44^,^45^ Indeed, it is thought that the initial step in a heterocondensation reaction between two metal precursors M and M′ involves ligand exchange and can be written as:

3 MCl_*n*_ + M′(OR)_*n*_ → MCl_*n*–*x*_(OR)_*x*_ + M′Cl_*x*_(OR)_*n*–*x*_,

Evidence for such ligand-exchange comes from direct observations of chloroalkoxides by Vioux^14^,^36^ as well as the following observations derived from nanocrystal synthesis: (a) reactions of HfCl_4_ and Zr(OR)_4_ and ZrCl_4_ and Hf(OR)_4_ yield exactly the same compositions of solid-solution Hf_*x*_Zr_1–*x*_O_2_ nanocrystals suggesting that ligand scrambling precedes condensation;^37^ (b) the reaction of trivalent lanthanide chlorides and lanthanide alkoxides yields lanthanide oxyhalide nanocrystals with well-defined oxyhalide bridges.^41^–^43^ The rates of condensation of the chloroalkoxide species produced as per eqn (3) are greatly dependent on the specific metal; precursors with similar reactivities yield solid-solution nanocrystals with a random distribution of two metals, whereas if one species reacts much faster than the second, very little of the second species incorporates within the lattice and the oxide of the first metal is obtained as the primary product.^36^ Reasonably well-matched precursor pairs such as Hf/Zr and La/Ce yield solid-solution nanocrystals of Hf_*x*_Zr_1–*x*_O_2_ and La_*x*_Ce_1–*x*_O_2_, respectively, albeit with compositions that reflect the relative reactivities of the two metal precursors as modified by the alkoxide ligands.^18^,^37^,^39^ However, upon reacting Hf precursors with Ce and La precursors, the homocondensation of the former proceeds much faster than heterocondensation. Indeed, HfO_2_ with very low dopant concentrations is obtained as the exclusive product upon the reaction of HfCl_4_ with mixtures of Hf(O^*t*^Bu)_4_ and Ce(O^*t*^Bu)_4_/La(O^i^Pr)_3_. The relatively low incorporation of Ce and La within the doped HfO_2_ lattice, very much lower than the precursor concentrations, corroborates the idea of the lower reactivity of these precursors. Fig. 1, 2, 4, and 5 nevertheless indicate that the addition of Ce(O^*t*^Bu)_4_ and La(O^i^Pr)_3_ allows for substantial tunability of the size of the obtained doped HfO_2_ nanocrystals. In Scheme 1, the initial step involves the formation of an alkoxo-bridge as a result of a Lewis acid–Lewis base interaction between the oxygen atom of the alkoxide and the metal center of a halide. Subsequently, the MX_3_OR species (derived from ligand exchange as per eqn (3)) mediates conversion of the alkoxo-bridge to an oxo-bridge with elimination of an alkyl halide. Indeed, Vioux has shown that the presence of MX_3_OR species is imperative for condensation and that MX_2_(OR)_2_ and MX(OR)_3_ species are much less reactive.^36^ In other words, the Lewis acid–base complex depicted in Scheme 1 is the “monomer”, whereas the MX_3_OR species formed by ligand exchange serves as the catalyst. The retention of equimolar amounts of halide and alkoxide precursors ensures that the amount of catalyst is kept essentially the same for all of the reactions listed in Tables 1 and 2 as a result of the ligand exchange reaction depicted in eqn (3) although it must be qualified that the equilibrium constants for formation of the catalyst will be altered for the different alkoxides. Indeed, since formation of an oxo-bridge, and not diffusion of monomers, is thought to be the rate-determining step, in the absence of a sufficient amount of the catalyst, such as at low alkoxide concentrations, only amorphous aggregates are obtained. However, since the Lewis acid–base adducts constituted from HfCl_4_ and Ce(O^*t*^Bu)_4_/La(O^i^Pr)_3_ are much less reactive as compared to those prepared from HfCl_4_ and Hf(O^*t*^Bu)_4_, the addition of cerium and lanthanum precursors essentially reduces the concentration of the active monomer. In other words, the reaction rate is diminished as a result of the lower reactivity of mixed metal Lewis acid–base adducts towards formation of oxo-bridges. As further corroboration for this idea, Table 1 and Fig. 1 indicate that the homocondensation of hafnium precursors yields pure HfO_2_ nanocrystals that are 12.9 ± 1.8 nm. In contrast, the homocondensation of cerium precursors yields CeO_2_ nanocrystals that are 1.5 ± 0.5 nm indicating much slower kinetics of growth (Table 1 and Fig. S2†). Cross-reactions between La and Ce alkoxides indicate even lower reactivities for the La precursors with only *ca.* 20 at% incorporation of La within solid-solution La_*x*_Ce_1–*x*_O_2_ nanocrystals when starting with equimolar concentrations.^39^ The reduced concentration of the active monomer brings about a systematic and pronounced diminution in the size of the doped HfO_2_ nanocrystals by inhibiting their growth. In other words, the cross-condensation reaction enables modulation of the concentration of the active monomer, thereby enabling precise control over crystal size.

**Fig. 7:**
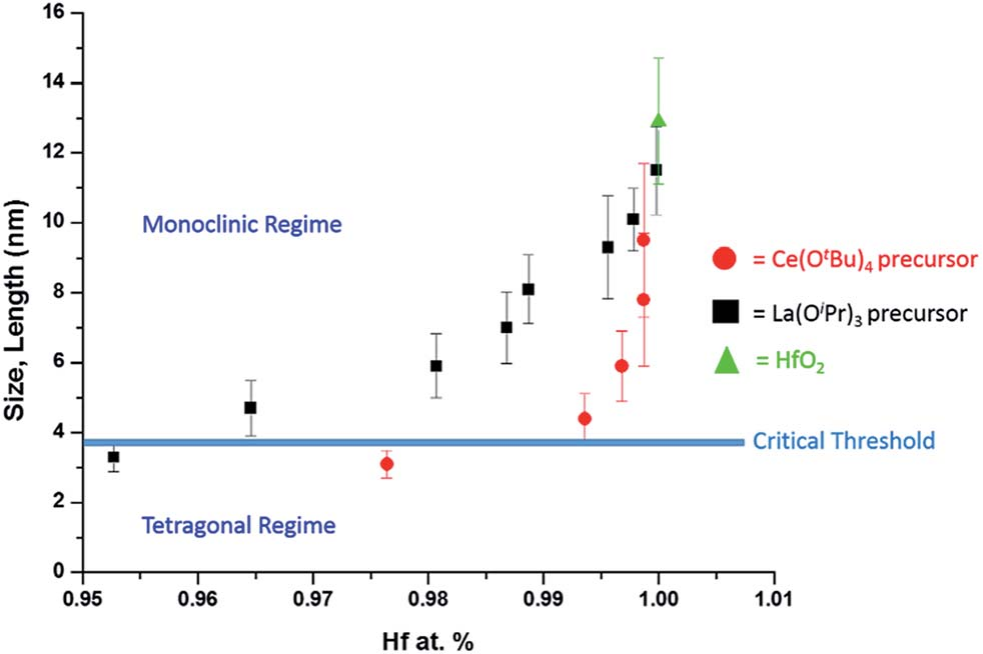
A plot of nanocrystal size *versus* the relative atomic percentage of Hf detected by elemental analysis (Tables 1 and 2). The line denoted as the critical threshold separates the monoclinic and tetragonal phases of pure HfO_2_.

As a next point, we discuss the origins of the stabilization of the tetragonal phase of HfO_2_. Indeed, the product of the direct reaction of HfCl_4_ and Hf(O^i^Pr)_4_ when ramped at a rate of 15 °C to 500 °C during thermal analysis is monoclinic HfO_2_, the thermodynamically stable phase, along with an oxygen-deficient orthorhombic HfO_2_ phase. The inclusion of TOPO still yields monoclinic HfO_2_, albeit with a smaller size. These results illustrate that direct reaction of the precursors cannot yield the metastable, kinetically trapped phase. The incorporation of the La and Ce alkoxides is imperative to slow the kinetics of crystal growth. Fig. 7 indicates the evolution of size as a function of the measured Hf concentration in the doped nanocrystals and depicts that only below a critical threshold of *ca.* 3.6–3.8 nm is the tetragonal phase stabilized. Since Tables 1 and 2 indicate that some amount of La and Ce are incorporated within the doped nanocrystals, it is worth considering whether the stabilization of the tetragonal phase results from (a) the size of the La/Ce cations that are displacively doped being different from that of Hf-cations (a strain effect), (b) the creation of oxygen vacancies, or (c) crystal size. Based on the larger size of La^3+^ cations (110 pm for seven-coordinated and 116 pm for eight-coordinated sites) as compared to Ce^3+^ (107 pm for seven-coordinated and 114.3 pm for eight-coordinated) or Ce^4+^ (97 pm for eight-coordinated),^46^,^47^ if the effects of cation size were to be of paramount importance, one would expect that incorporation of La would more readily bring about stabilization of the tetragonal phase and one would further expect to see substantial tensile strain. However, Tables 1 and 2 indicate that the amount of detected Ce and La are 2.36 at% and 4.73 at%, respectively, for stabilization of the tetragonal phase and thus size of the cations is likely not the primary driving force for stabilization of the tetragonal phase. Furthermore, the reconstructed HAADF images (Fig. 3 and 6) do not reveal any measurable strain for the tetragonal structure. In other words, if size were the primary factor, relatively lower amounts of La-incorporation would be expected to bring about stabilization of the tetragonal phase (Table 2 indicates that the Hf_1–*x*_La_*x*_O_2_ nanocrystals remain monoclinic even upon incorporation of 3.54 at% of La).

The second scenario pertains to the potential role of oxygen vacancies. The substitutional incorporation of trivalent lanthanum cations in the HfO_2_ lattice will result in creation of half an oxygen vacancy to maintain electrostatic neutrality. The Ce^4+^/Ce^3+^ redox couple is readily accessible but since we start with tetravalent cerium precursors, the vacancy concentration generated upon cerium-incorporation is likely to be lower than upon lanthanum incorporation. Again, if oxygen vacancies were to provide the driving force for stabilization of the tetragonal phase, one would expect that lanthanum-incorporation should more readily bring about stabilization of the tetragonal phase as compared to cerium incorporation. Again, Tables 1 and 2 indicate that Hf_1–*x*_La_*x*_O_2_ nanocrystals with 3.54 at% La are monoclinic, whereas Hf_1–*x*_Ce_*x*_O_2_ nanocrystals with only 2.36 at% Ce are tetragonal. The preceding discussion and Fig. 7 thus implies that it is the size of the nanocrystals that is the primary driving force for stabilization of the tetragonal phase. The stabilization of this phase is derived from the surface energy of the tetragonal phase being lower than that of the monoclinic phase (the difference between the two phases is 246 mJ m^–2^).^21^ Below the critical size regime, the surface-to-volume ratio of pure HfO_2_ nanocrystals becomes such that the surface energy term exceeds the bulk energy component of the free energy term (the difference between the two phases is 196 meV for pure HfO_2_),^21^ thereby enabling preferentially stabilization of the tetragonal phase at room temperature.^1^,^2^,^8^,^9^

In 1972, Bailey and co-workers developed an expression for calculating the critical size for stabilizing a metastable state based on a classical thermodynamic treatment of the phase transformation and the competing bulk and surface energy terms. In this formulation, the critical size, *d* can be expressed as^48^
4

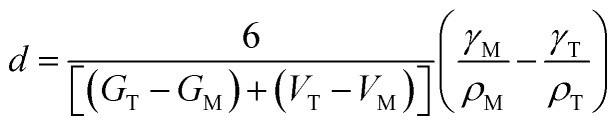

where the subscripts M and T correspond to the relevant values for the monoclinic and tetragonal phases, *G* is the volume free energy change across the phase transformation, *V* is the strain energy, *γ* is the specific surface energy, and *ρ* represents the density. Hunter *et al.* used this expression to predict a critical size for stabilization of tetragonal HfO_2_ of *d* = 3.6 nm.^16^ In the absence of well-defined, monodisperse, and phase-pure nanocrystals, a comprehensive evaluation of this formalism has not been possible thus far. Another notable complication for HfO_2_ nanocrystals prepared by aqueous methods is the almost 20–30% diminution of surface energy as a result of surface hydroxylation, which in this case further dilutes the influence of the surface energy contribution.^20^Fig. 7 indicates a critical size of 3.6–3.8 nm for stabilization of the tetragonal phase of HfO_2_, which is in remarkably good agreement with predictions from this thermodynamic model. The origin of the stability of the tetragonal phase at these dimensions can thus be attributed to the substantially lower surface energies of this phase, which renders this phase energetically stable under conditions of constrained equilibrium.

## Conclusions

Stabilization of the tetragonal phase of HfO_2_ has been far more challenging as compared to ZrO_2_ given the smaller volume expansion accompanying the tetragonal → monoclinic transformation and the relatively greater stabilization enjoyed by the monoclinic phase. Classical thermodynamic models predict that the tetragonal phase should be stable at dimensions smaller than *ca.* 3.6 nm but the validity of these predictions have been thus far impossible to determine in the absence of synthetic approaches that can access such ultra-small dimensions with precise control of particle size. In this work, we have developed a non-hydrolytic condensation route wherein the concentration of the active monomer is precisely modulated by replacing Hf(O^*t*^Bu)_4_ with less reactive Ce(O^*t*^Bu)_4_ or La(O^i^Pr)_3_ precursors. The latter alkoxides exhibit much slower kinetics of condensation and thus are incorporated within the doped HfO_2_ lattice only to small extents but play a significant role in modifying the growth kinetics by suppressing the concentration of the active monomer. This approach enables precise control of size in ultra-small dimensions and allows for systematic evaluation of the size-dependence of phase stabilities in this system. The much desired metastable tetragonal phase is stabilized at dimensions less than 3.6–3.8 nm, which is in good accord with predictions of thermodynamic models that take into account the competing influences of bulk free energy and specific surface energy.

## Conflict of interest

The authors declare no competing financial interest.

## Supplementary Material

Supplementary information
