# Supplementary material for: Stabilizing metastable tetragonal HfO2 using a non-hydrolytic solution-phase route: ligand exchange as a means of controlling particle size
Source: Chem Sci. 2016 May 3;7(8):4930–9. doi: 10.1039/c6sc01601d (PMC6018514; doi:10.1039/c6sc01601d)
Supplement: Supplementary file 1 [file SC-007-C6SC01601D-s001.pdf]

Electronic Supplementary Material (ESI) for Chemical Science.
This journal is © The Royal Society of Chemistry 2016

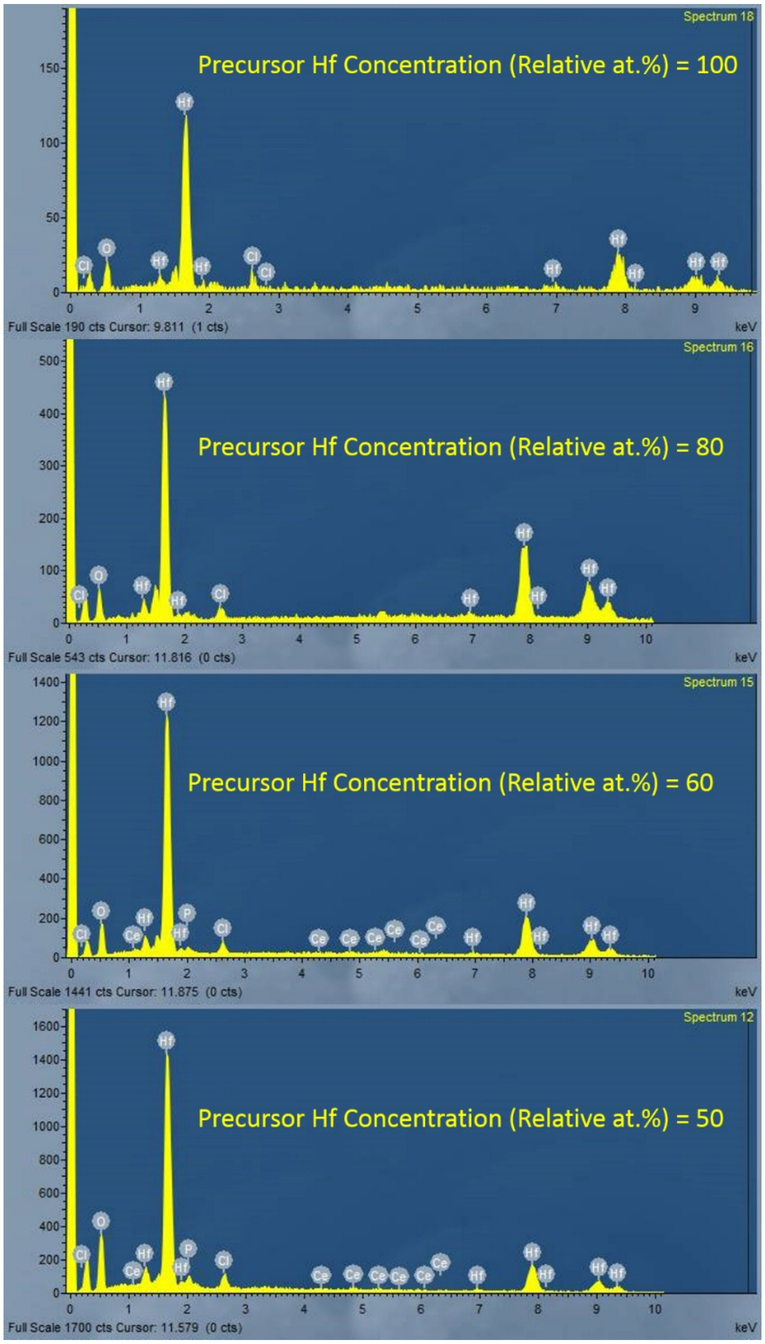

Figure S1. Energy dispersive X-ray spectroscopy (EDX) of pristine HfO_2 and selected $Hf_{1-x}Ce_xO_2$ nanocrystals. The detected concentrations of hafnium and cerium are listed in Table S1.

Precursor Hf Concentration (Relative at.%)	Detected Hf Concentration by EDX (Relative at. %)	Detected Ce Concentration by EDX (Relative at. %)
100	100	0
80	100	Not Detected
60	99.75	0.25
50	96.13	3.87

Table S1. Detected concentrations of hafnium and cerium by EDX analysis based on integration of the data shown in Figure S1.

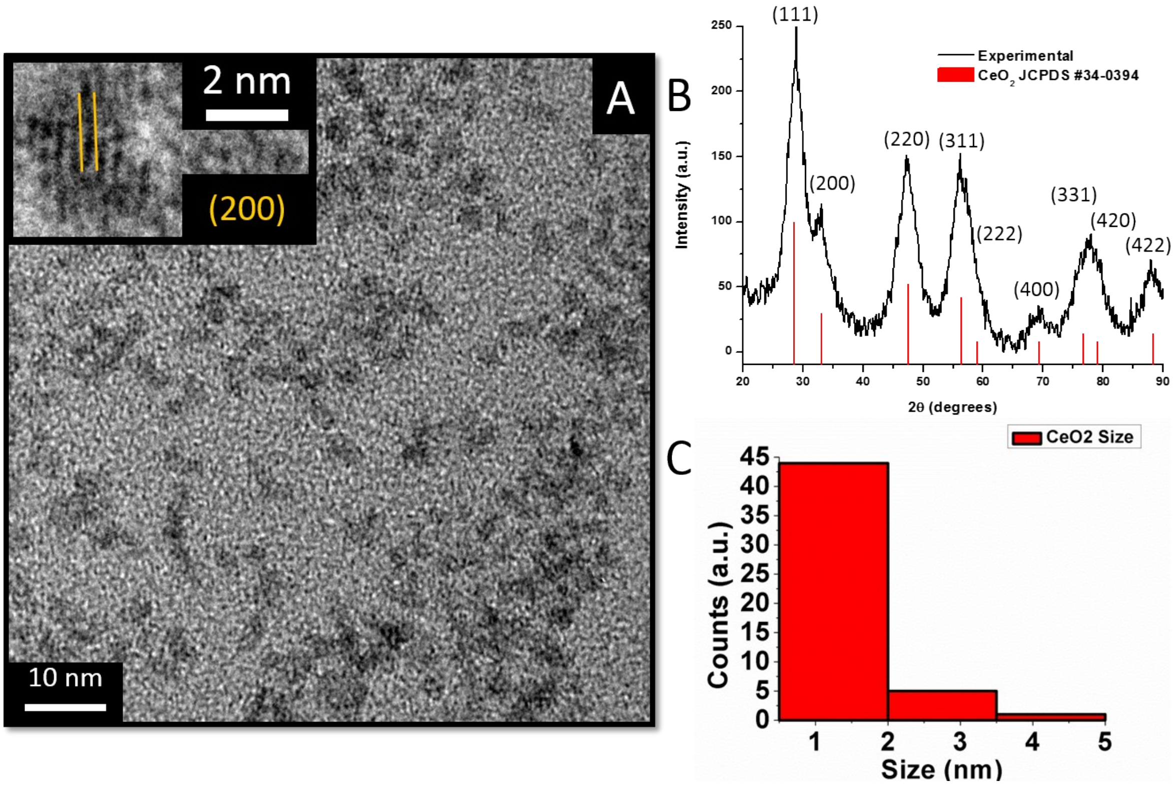

Figure S2. (A) Low-magnification transmission electron microscopy image of CeO_2 nanocrystals. The inset shows a HRTEM image indicating the separation between the (200) lattice planes of the cubic phase. (B) X-ray diffraction pattern of CeO_2 nanocrystals synthesized using 2 mmol of $CeCl_3$ and 2 mmol of $Ce(O^tBu)_4$. Reflections of cubic CeO_2 are indicated in red (JCPDS # 34-0394). (C) Size distribution histogram indicating the size of the CeO_2 nanoparticles to be ca. 1.5 ± 0.5 nm.

HfO_2 (Monoclinic) – JCPDS # 78-0050				
atom	*x*	*y*	*z*	occupancy
Hf1	0.2755(2)	0.0397(1)	0.2080(2)	1.0
O1	0.0739(20)	0.3318(17)	0.3466(17)	1.0
O2	0.4489(20)	0.7582(16)	0.4800(22)	1.0
Space Group = $P2_1/c$, *a* = 5.1170(1), *b* = 5.1754(2), *c* = 5.2915(2) α, γ = 90, β= 99.216(2)				
HfO_2 (Tetragonal) – From Ref. 40				
atom	*x*	*y*	*Z*	occupancy
Hf1	0.0	0.0	0.0	1.0
Hf2	0.5	0.5	0.5	1.0
O1	0.5	0.0	0.21282	1.0
O2	0.5	0.0	-0.28718	1.0
O3	0.0	0.5	0.28718	1.0
O4	0.0	0.5	-0.21282	1.0
Space Group = $P4_2/nmc$, *a, b* = 3.560000, *c* = 5.110000, α, β, γ = 90				

Table S2. Unit cell parameters for the monoclinic and tetragonal phases of HfO_2.

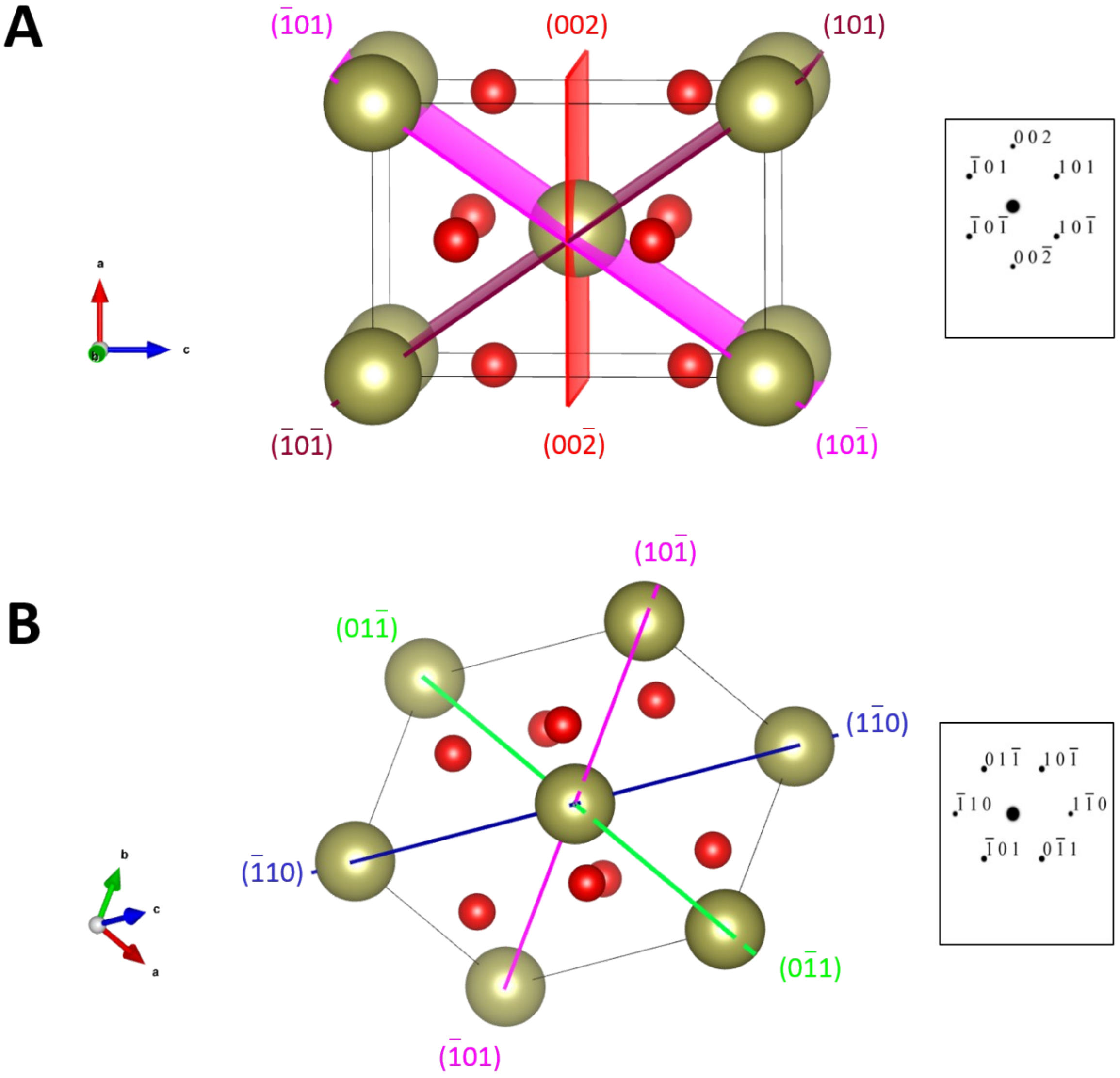

Figure S3. (A) HfO_2 tetragonal unit cell depicting the corresponding lattice planes from Figure 3D. (B) HfO_2 tetragonal unit cell depicting the corresponding lattice planes from Figure 3H. The unit cells were constructed using the lattice parameters given in Ref. 40.

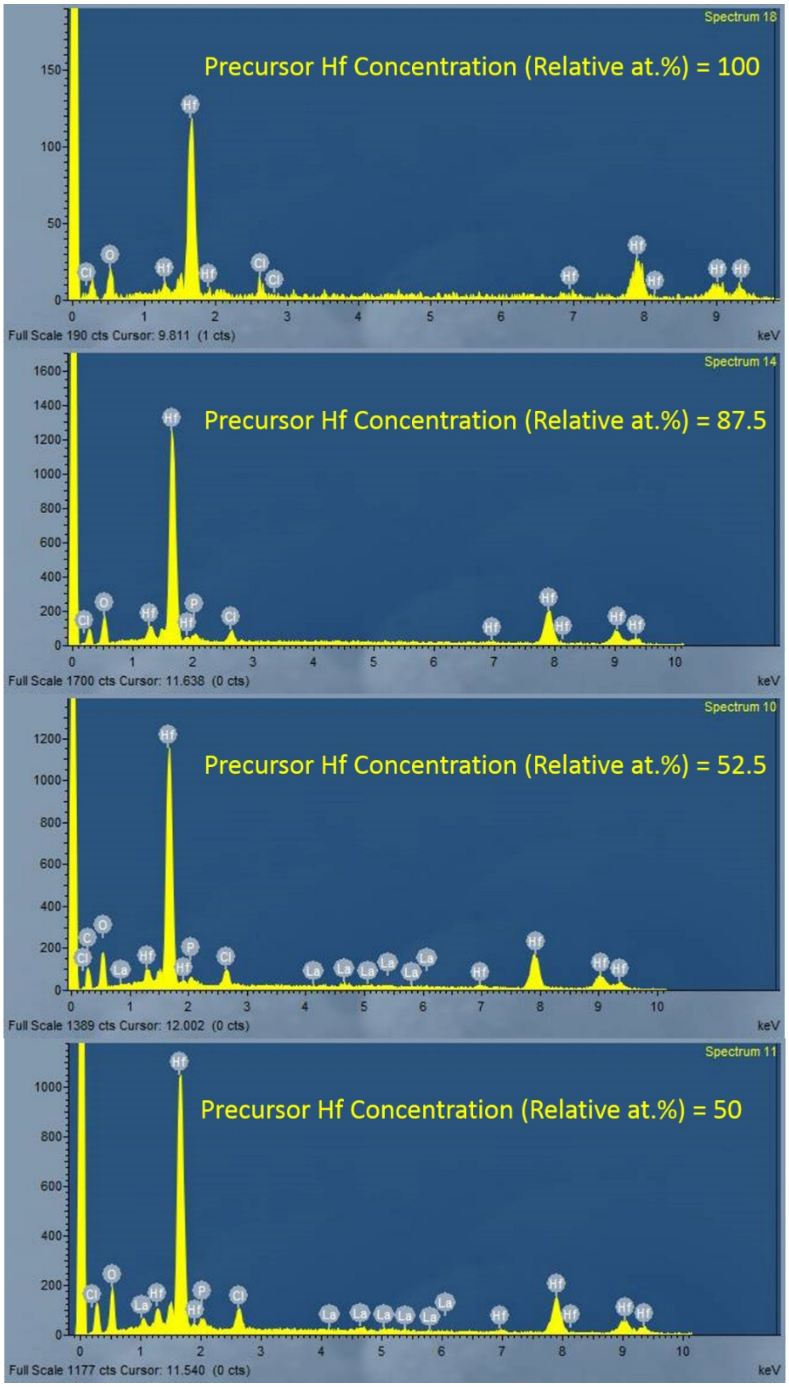

Figure S4. Energy dispersive X-ray spectroscopy (EDX) of pristine HfO_2 and selected $Hf_{1-x}La_xO_2$ nanocrystals. The detected concentrations of hafnium and lanthanum are listed in Table S3.

Precursor Hf Concentration (Relative at.%)	Detected Hf Concentration by EDX (Relative at. %)	Detected La Concentration by EDX (Relative at. %)
100	100	0
87.5	100	Not Detected
52.5	96.81	3.19
50	95.99	4.01

Table S3. Detected concentrations of hafnium and lanthanum by EDX analysis integrated from the spectra depicted in Figure S4.

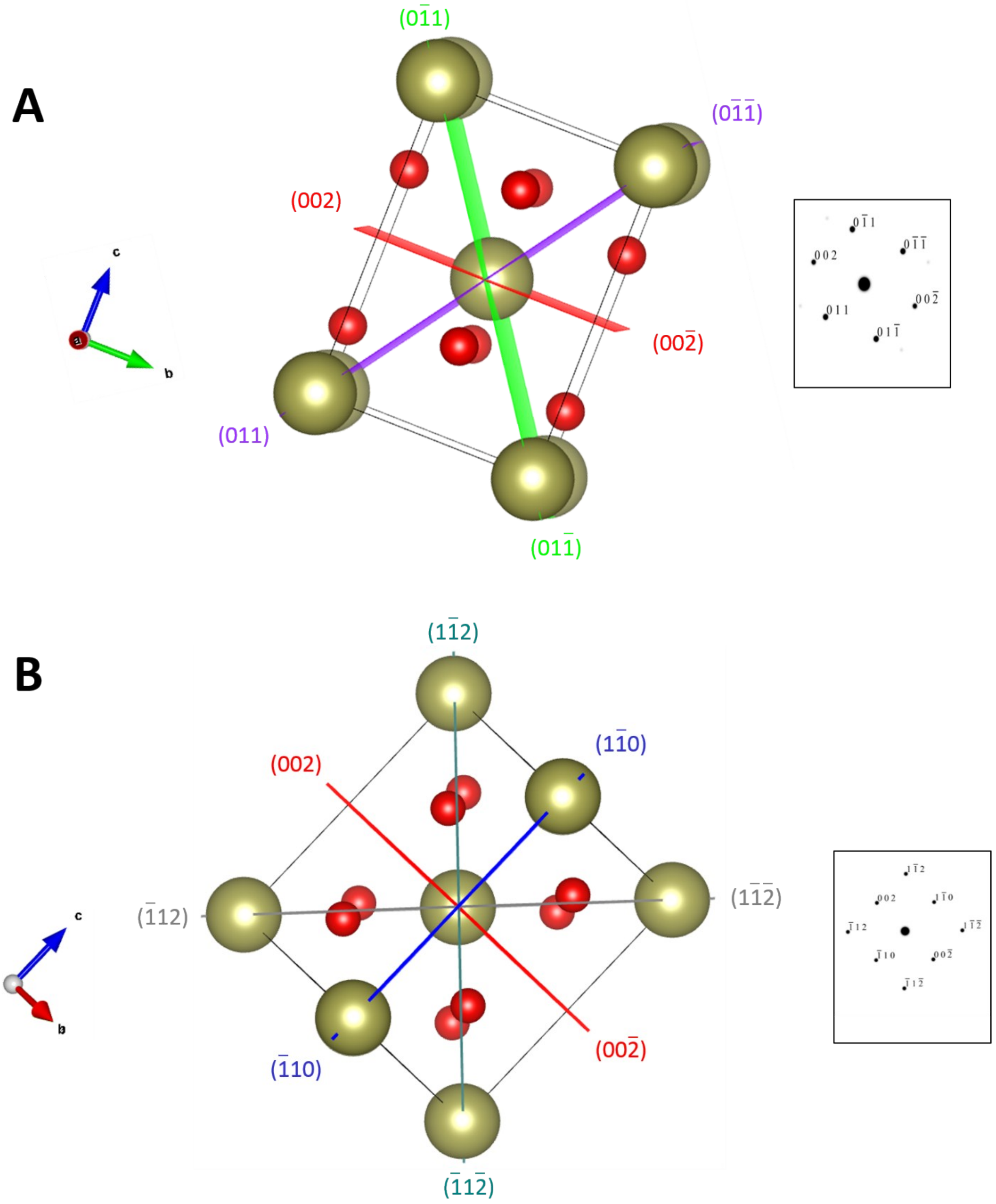

Figure S5. (A) HfO_2 tetragonal unit cell depicting the corresponding lattice planes from Figure 6D. (B) HfO_2 tetragonal unit cell depicting the corresponding lattice planes from Figure 6H The unit cells are constructed using lattice parameters given in Ref. 40.
